# Comparing outcomes of right verse left hepatic approach percutaneous biliary drainage catheters^☆^

**DOI:** 10.1016/j.sopen.2024.05.014

**Published:** 2024-05-29

**Authors:** Brent Smith, Jodi Veach, Carissa Walter, Alexander Alsup, Kate Young, Lauren Clark, Yanming Li, Aaron Rohr

**Affiliations:** a Department of Radiology, University of Kansas Medical Center, 4000 Cambridge Street, Kansas City, KS, USA; b University of Kansas School of Medicine, 3901 Rainbow Blvd, Kansas City, KS, USA; c Department of Biostatistics & Data Science, University of Kansas Medical Center, 4000 Cambridge Street, Kansas City, KS, USA

**Keywords:** Biliary drainage catheters, Right verse left hepatic approach, Biliary drain, Hepatobiliary drain, Cholangitis

## Abstract

**Purpose:**

Determine if there is a difference in adverse events (AE) between right or left hepatic percutaneous biliary drain placement (PTBD) in patients with biliary strictures.

**Materials & methods:**

This retrospective study included patients with benign or malignant biliary stricture treated with PTBD at a single institution from 7/28/2004–3/30/2021. 357 patients met inclusion criteria, 77 (21.6 %) had PTBD on the left and 280 (78.4 %) on the right. AEs associated with the initial drain placement or during subsequent intervention were collected and categorized. AEs that were grouped as periprocedural included: surgery, infection, hemorrhage, and drain failure. AEs in the postprocedural group included: chills, catheter displacement, cholangitis, biliary stones, drain malfunction, fever resolving without treatment, and pericatheter leakage. Surgery was considered a major AE and the remaining AEs were categorized as minor. Statistical analyses were performed using Logistic Regression Analysis and *p*-values less than 0.05 were considered statistically significant.

**Results:**

Overall, there was no statistically significant difference in AEs between right and left drains in the periprocedural and postprocedural period (*p* = 0.832, OR = 0.95 and *p* = 0.808, OR = 0.93 respectively). When analyzing minor AEs individually, only cholangitis occurred at a higher rate on the right side (*p* = 0.033, OR = 0.43). There was no statistical difference in the rate of major AEs in the periprocedural period between left and right drains (*p* = 0.311, OR = 1.37).

**Conclusion:**

Current literature is equivocal when comparing right versus left percutaneous biliary drains. This analysis describes no statistically significant difference in AEs between right and left hepatobiliary drains aside from slightly higher incidence of cholangitis for right sided drains.

## Introduction

Biliary stricture is an unfortunate clinical diagnosis and often treated by Interventional Radiologists. Benign biliary strictures may be secondary to several etiologies including trauma, cholelithiasis, pancreatitis, postprocedural and inflammatory disorders [1]. Malignant biliary strictures are most frequently secondary to primary pancreaticobiliary malignancies, though lymphoma and metastatic disease are also potential causative tumors [2,3]. Percutaneous transhepatic cholangiography (PTC) and percutaneous transhepatic biliary drainage (PTBD) are common procedures performed for diagnosis and treatment of both malignant and benign hepatobiliary disorders [1]. When surgical or endoscopic therapies are suboptimal or not feasible, using fluoroscopic guided PTBD with possible drain or stent placement is a reasonable treatment option. Furthermore, as institutions continually adopt a benign biliary stricture treatment protocols (BBSP), PTBD will continue to be a mainstay therapy option for patients with biliary disease [10].

While benign and malignant etiologies may result in peripheral stricture specifically requiring a right or left drain, obstruction at or below the level of the biliary confluence may be treated with either approach. A specific example of laterality of drain being predetermined is a case of hilar cholangiocarcinoma that is resectable via hemipatectomy; in this situation, there is evidence to support selectively draining the future remnant liver rather than the side being resected. Previous studies have shown unilobar hepatic drainage to be equally effective as bilobar drainage with obstruction at the confluence [4] and suggest relieving just 30 % of the hepatic parenchyma is sufficient to reduce symptoms of obstructive jaundice [5,6]. These factors currently contribute to drain laterality often being selected based on the patients' personal preference (e.g. intercostal pain, ease of maintenance or concealment) rather than objective clinical parameters such as procedural complication rate or risk of subsequent drain failure. Long term maintenance for biliary drains are largely dependent on the institution. Typically, drains will be followed until either removal or permanency. The degree of which varies widely, depending on whether the placement is for benign, malignant, transient process, iatrogenic, or a different reason which determines the ultimate long-term course. Retrospective studies comparing right versus left approach PTBD are scarce with the current research comprised primarily of prospective studies that are limited in their long-term evaluation of drain adverse events (AE) and evaluate only malignant or benign strictures. Moreover, there is no current consensus on which approach is technically safer; one study suggested that the right approach carried an increased risk of bleeding [7], while others suggested the left approach [8,9] or have found no statistically significant difference whatsoever [2]. This study aims to compare the periprocedural, postprocedural AE incidence and additional surgical intervention rates between right and left PTBD with additional long-term evaluations.

## Materials and methods

Following institutional review board approval, patient medical records were reviewed in compliance with Health Care Portability and Accountability Act guidelines. Patients who received PTBD for treatment of benign and malignant biliary stricture were identified using Picture Archiving and Communications System (PACS) integrated data mining software (Illuminate Insight; Softek, Kansas City, Kansas). Study data was then managed using REDCap (Research Electronic Data Capture) electronic data capture tools.

Patients with benign stricture underwent repeat intervention based on the institution's BBSP or at the discretion of the interventional radiologists, depending on the patients change in status requiring reintervention. Patients with malignant stricture received palliative treatment with routine drain exchanges at set intervals; however, both groups underwent repeat intervention when the patient demonstrated clinical signs of drain failure. All procedures were performed by fellowship-trained interventional radiologists with an average of 10 years of experience (range 2–22 years).

Patients were followed from the time of initial biliary drain placement to the time of death, or last available note in the electronic medical record (median = 342 days, IQR = 67.5–1446.2 days). The primary analysis grouped AEs occurring in intervention related periprocedural period versus the treatment related postprocedural period, which were further classified as minor or major. The periprocedural period consisted of intraprocedural, immediately after, and any required short-term hospitalization associated with the drain placement. The postprocedural period was quantified for any time thereafter. Among periprocedural associated AEs, only emergent surgical intervention was classified as a major AE. Minor periprocedural associated AEs included infection, hemorrhage, and drain failure. Among postprocedural associated AEs, no AEs were classified as a major. Minor postprocedural AEs included chills, catheter displacement, cholangitis, biliary stones, drain malfunction, fever resolving without treatment, and pericatheter leakage. Any clinical, laboratory, or radiologic evidence was used to evaluate for a malfunctioning drain.

The analysis was conducted using version 4.1.1 of the R statistical programming software. Univariate logistic regression models were employed to examine the association with biliary catheter laterality and the occurrence of adverse events. The outcome variables for each model were binary, with “No Complications” as the reference group compared to “Any Complications” (including major and minor complications) or “Major Complications” (major complications only). We utilized the ‘glm()’ function in R with the default Wald test for hypothesis testing. The models were specified with the binomial family to account for the binary nature of the outcome variable. Model goodness-of-fit was assessed using standard techniques, including the Hosmer-Lemeshow test for logistic regression models. The significance level for all statistical tests was set at alpha = 0.05.

## Results

This retrospective study included all patients diagnosed with benign or malignant biliary stricture who underwent PTBD in the interventional radiology division at a single institution from July 2004 to March 2021. Patients were excluded if they initiated or completed percutaneous therapy at an outside institution or had bilateral biliary drains placed. Three hundred fifty-seven patients met inclusion criteria, with 77 (21.6 %) utilizing drains on the left and 280 (78.4 %) on the right, with a mean age of 65.1 years. See Table 1.

**Table 1:** Demographics.

Characteristic	Left *n* = 77 (21.6 %)	Right *n* = 280 (78.4 %)	P-value
Age, mean (SD)	63.4 (15.7)	65.9 (14.0)	0.219
Sex, n(%)			0.058
Female	39 (50.6)	106 (37.9)	
Male	38 (49.4)	174 (62.1)	
Race, n(%)			0.826
White	65 (84.4)	232 (82.9)	
Black	5 (6.5)	17 (6.1)	
Asian/Pacific Islander	1 (1.3)	5 (1.8)	
American Indian	1 (1.3)	1 (0.4)	
Unknown	5 (6.5)	25 (8.9)	
Ethnicity, n(%)			0.479
Non-Hispanic/Latino	73 (94.8)	261 (93.2)	
Hispanic/Latino	2 (2.6)	15 (5.4)	
Unknown	2 (2.6)	4 (1.4)	

The rate of no AE in the intervention related periprocedural period was similar between left (50.6 %) and right (49.6 %) hepatic approach drains with logistical regression analysis demonstrating no statistical significance (*p* = 0.832, OR = 0.95). Furthermore, the rate of no AE in the treatment related postprocedural period was also similar for left (36.4 %) and right (37.5 %) hepatic approach drains (*p* = 0.808, OR = 0.93). When individual minor AEs were evaluated, there was no statistically significant difference between left and right drains for any minor AEs in the periprocedural period. However, when individual AEs in the postprocedural treatment period were analyzed individually there was a statistically significant difference in the rate of cholangitis between left sided drains (10.4 %) and right sided drains (21.4 %) (*p* = 0.033, OR = 0.43). In the periprocedural period, there was no statistically significant difference in the rates of surgery between left (23.4 %) and right drains (18.2 %) (*p* = 0.311, OR = 1.37). See Table 2.

**Table 2:** Perioperative and postoperative period AE rates.

Complication Type	Left n = 77 n (%)	Right n = 280 n (%)	Odds Ratio	95 % CI	Wald-Test P-value
Intervention perioperative period AEs					
None	39 (50.6)	139 (49.6)	0.95	(0.57,1.56)	0.832
Minor AEs					
Infection	22 (28.6)	84 (30.0)	0.93	(0.54,1.63)	0.808
Bleed	8 (10.4)	29 (10.4)	1.00	(0.44,2.30)	0.993
Drain failure	1 (1.3)	10 (3.6)	0.36	(0.05,2.82)	0.328
Major AEs					
Surgery	18 (23.4)	51 (18.2)	1.37	(0.75,2.52)	0.311
Treatment postoperative period AEs					
None	28 (36.4)	105 (37.5)	0.93	(0.54,1.63)	0.808
Minor AEs					
Chills	7 (9.1)	24 (8.6)	1.07	(0.44,2.58)	0.886
Catheter displacement	7 (9.1)	33 (11.8)	0.75	(0.32,1.77)	0.508
Cholangitis	8 (10.4)	60 (21.4)	0.43	(0.19,0.93)	0.033
Biliary stones	1 (1.3)	6 (2.1)	0.60	(0.07,5.07)	0.640
Drain malfunction	9 (11.7)	38 (13.6)	0.84	(0.39,1.83)	0.666
Fever resolving without treatment	4 (5.2)	6 (2.1)	2.50	(0.69,9.10)	0.164
Pericatheter leakage	21 (27.3)	65 (23.2)	1.24	(0.70,2.20)	0.461

Lastly, there was no difference in odds or having major vs none nor any complications vs none between left and right drains. See Table 3.

**Table 3:** Odds ratios between left and right drains.

Intervention perioperative period AEs	OR	95 % CI	Wald-Test P-value
Major (surgery) vs None	1.26	(0.66,2.40)	0.485
Any Complication vs None	0.89	(0.58,1.59)	0.876
Treatment postoperative period AEs			
Any Complication vs None	0.86	(0.62,1.77)	0.855

## Discussion

This retrospective study included patients with benign or malignant biliary stricture treated with PTBD and evaluated adverse events regarding the periprocedural and postprocedural periods. There were no statistically significant differences in major AEs between right and left drains in the periprocedural and postprocedural period. When considering minor AEs individually, cholangitis in the postprocedural period was the sole AE that occurred at a statistically significant different rate between left and right sided drains. There was no difference in odds or having major vs none nor any complications vs none between left and right drains.

**Fig. 1:**
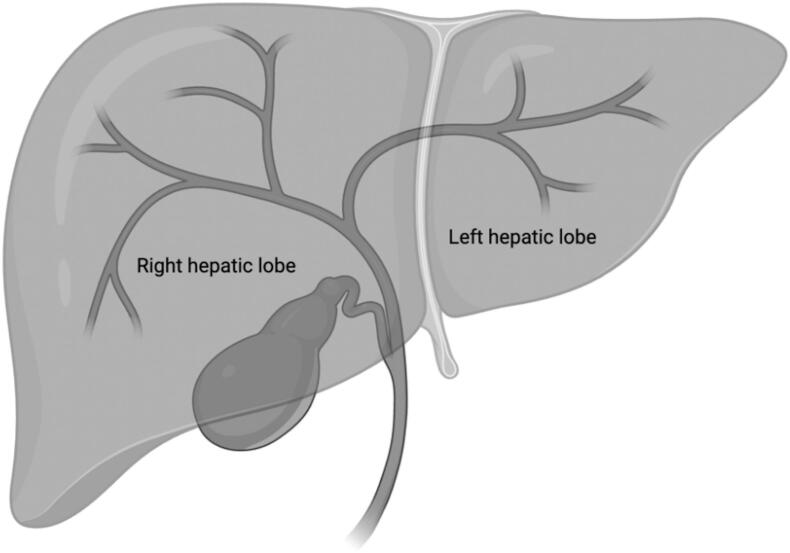
Typical intrahepatic biliary branching pattern demonstrates the right and left hepatic lobe. Created with Biorender.com

This study suggests there may be greater chance of developing cholangitis during the postprocedural period with right hepatic approach biliary drains but found no difference in any other AEs between left and right approaches. There is no current, available literature that corroborates this correlation between right sided interventions and cholangitis, but the anatomy of the biliary tree may provide further insight. The right hepatic duct is generally much shorter and bifurcates earlier than the left hepatic duct, measuring 0.9 cm on the right versus 3.0 cm on the left [5]. The left hepatic duct also generally branches at a more acute angle from the common hepatic duct than the much more obtuse or streamlined right hepatic duct, see Fig. 1. This difference in branch angle and length of the duct prior to bifurcation may cause the progression of infection or inflammation along the right hepatic more frequently than the left hepatic duct.

Current literature regarding efficacy and safety of right versus left percutaneous biliary drains is equivocal and each side may have its advantages and disadvantages. Prior studies have been limited due to prospective small group analysis or limiting patient populations strictly to malignant or benign biliary strictures. Nonetheless, the consensus favors right hepatic approach drains even though there is research that favors left biliary drains due to decreased bleeding [7]. Frequently, radiologists may choose whichever ductal system is the most dilated allowing for a less complicated placement, but often the laterality is purely at the discretion of the provider. The decision to place a right versus left percutaneous biliary drain is universally operator dependent. Many technical and physical challenges are rationalized when making this decision. They vary from patient tolerability (i.e. intercostal versus peritoneal), isolated biliary systems on imaging, degree of dilation, future planned surgeries, etc. However, right-sided percutaneous biliary drains are generally easier for wound management and maintenance flushes.

There are several limitations to this study. Patients were not randomized to left or right drains and provider preference and variance in technique may have introduced bias. The study group also revealed asymmetry in terms of number of left versus right biliary drains included. This is likely due to a subjective preference of providers who placed the drains in this single institution study. AE data was captured retrospectively, limiting the ability to properly standardize or catch all instances of AEs. Additionally, some patients may have had follow-up or received treatment for AEs at outside institutions, and these instances would not have been captured in this data set. Finally, there is a potential for bias due to provider preference for right versus left biliary drains in patients where either side was suitable.

This retrospective analysis describes no statistically significant difference in AEs between right and left hepatobiliary drains aside from slightly higher incidence of cholangitis for right sided drains. Future research including a randomized controlled trial may further explore this finding. Current research suggests providers may continue to choose either a right or left sided drain based on their own or the patients' preference.

## Ethical approval

This investigation has received ethical approval from the Institutional Review Board under protocol number 146534. The proposed study methodology, objectives, and potential risks to ethical principles and standards have been carefully reviewed and approved. There are no funding sources to disclose.

## CRediT authorship contribution statement

**Brent Smith:** Writing – review & editing, Writing – original draft, Methodology, Investigation, Formal analysis, Conceptualization. **Jodi Veach:** Writing – review & editing, Writing – original draft, Formal analysis, Data curation. **Carissa Walter:** Writing – review & editing, Writing – original draft, Methodology, Data curation, Conceptualization. **Alexander Alsup:** Formal analysis. **Kate Young:** Formal analysis. **Lauren Clark:** Formal analysis. **Yanming Li:** Formal analysis. **Aaron Rohr:** Writing – review & editing, Writing – original draft, Methodology, Investigation, Conceptualization.

## Declaration of competing interest

All authors declare that they have no conflict of interests.
